# Increased Activity Imbalance in Fronto-Subcortical Circuits in Adolescents with Major Depression

**DOI:** 10.1371/journal.pone.0025159

**Published:** 2011-09-16

**Authors:** Qing Jiao, Jun Ding, Guangming Lu, Linyan Su, Zhiqiang Zhang, Zhengge Wang, Yuan Zhong, Kai Li, Mingzhou Ding, Yijun Liu

**Affiliations:** 1 Department of Medical Imaging, Nanjing Jinling Hospital, Medical School of Nanjing University, Nanjing, China; 2 Department of Child Psychiatry, Mental Health Institute, The Second Xiangya Hospital of Central South University, Changsha, China; 3 Department of Radiology, Taishan Medical University, Taian, China; 4 Mental Health Center of Shenzhen, Shenzhen Kangning Hospital, Shenzhen, China; 5 Department of Pharmacology, Suzhou University, Suzhou, China; 6 Department of Biomedical Engineering, University of Florida, Gainesville, Florida, United States of America; 7 Department of Psychiatry and McKnight Brain Institute, University of Florida, Gainesville, Florida, United States of America; Federal University of Rio de Janeiro, Brazil

## Abstract

**Background:**

A functional discrepancy exists in adolescents between frontal and subcortical regions due to differential regional maturational trajectories. It remains unknown how this functional discrepancy alters and whether the influence from the subcortical to the frontal system plays a primacy role in medication naïve adolescent with major depressive disorder (MDD).

**Methodology/Principal Findings:**

Eighteen MDD and 18 healthy adolescents were enrolled. Depression and anxiety severity was assessed by the Short Mood and Feeling Questionnaire (SMFQ) and Screen for Child Anxiety Related Emotional Disorders (SCARED) respectively. The functional discrepancy was measured by the amplitude of low-frequency fluctuations (ALFF) of resting-state functional MRI signal. Correlation analysis was carried out between ALFF values and SMFQ and SCARED scores. Resting brain activity levels measured by ALFF was higher in the frontal cortex than that in the subcortical system involving mainly (para) limbic-striatal regions in both HC and MDD adolescents. The difference of ALFF values between frontal and subcortical systems was increased in MDD adolescents as compared with the controls.

**Conclusions/Significance:**

The present study identified an increased imbalance of resting-state brain activity between the frontal cognitive control system and the (para) limbic-striatal emotional processing system in MDD adolescents. The findings may provide insights into the neural correlates of adolescent MDD.

## Introduction

Adolescent MDD is associated with significantly high risk of suicide and the adolescent-onset depression is likely to have recurrent episodes of depression in adult life [1], [2], [3]. A better understanding of the functional differences among neural systems underlying cognitive-affective processing in adolescents with MDD is highly necessary. It may provide insights into prevention and treatment for this debilitating illness.

It has been hypothesized that the frontal-subcortical discrepancy of maturational trajectories may play a role in the increased risk for the development of affective disorders including adolescent MDD [4]. During development in adolescents, different brain regions follow distinct maturational trajectories, with the frontal cortex being one of the last brain structures to mature [5], [6]. In adolescents, while a number of frontal regions responsible for cognitive control are still under development, most of the subcortical regions (e.g., the basal ganglia, amygdala, nuclei accumbens) involved in affective processing have already achieved functional maturity [7], [8]. Thus, a functional discrepancy arises between (the frontal) cognitive control and (the subcortical) affective processing during adolescence, which is often characterized by enhanced bottom-up emotional drive or impulsivity but insufficiently developed top-down executive control under various task conditions [4], [8], [9].

Structural MRI studies have demonstrated MDD-related brain volume alterations in these cortical and subcortical regions, including reduced frontal white matter and smaller right caudate nucleus and rostral anterior cingulate cortices (ACC), and increased frontal gray matter and larger amygdale and hippocampal volume ratios [10], [11], [12], [13], [14]. Previous fMRI studies also reported abnormal activities in the same structures in association with mood induction and reward reinforcement in adolescent MDD [15], [16]. The interaction patterns between these brain regions are altered in adolescent MDD patients [1]. Using the subgenual ACC as a seed region, a resting-state fMRI connectivity analysis revealed decreased functional interconnection between the subgenual ACC and the other regions including the right medial frontal cortex, left frontal cortex, superior temporal gyrus, and the insular cortex in adolescent MDD [1].

It remains unclear, however, whether these structural and functional abnormalities give rise to exaggerated or diminished functional discrepancies between the emotion processing and cognitive control systems in the adolescent MDD. In the present study, we conducted a data-driven analysis of fMRI data from medication naïve MDD adolescents and healthy controls. As a cross-section study, the purpose of the present study lies in disclosing the alteration of resting-state activity of MDD patients under the fact of the brain functional imbalance between frontal and subcortical regions in adolescents, rather than investigating changes of the resting-state activity in the process of human development longitudinally. Our aim is focused on how the imbalance of brain activity between the emotional process system and the cognitive control system was altered in adolescents with MDD. To achieve these goals, the amplitude of low-frequency fluctuations (ALFF) of the blood oxygenation level-dependent (BOLD) fMRI signals was calculated to characterize the resting-state spontaneous brain activity levels and evaluate the activity level difference between the frontal and subcortical systems.

## Materials and Methods

### Subjects

Eighteen medication naïve unipolar depressed patients (see below Psychiatric evaluations for the diagnosis of MDD) aged from 13 to 17.5 years old (mean age ± SD = 15.8±1.2 years old, 10 female) and 18 age- and gender-matched healthy controls (mean age ± SD = 16.2±0.9 years old, 10 female) were recruited in the study. Ten of the MDD adolescents had psychiatric co-morbidity of anxiety disorders. Duration of illness in the MDD adolescents was shorter than six months and all of the patients were at their first onset.

Inclusion criteria of the adolescent patients included first episode, medication naïve of MDD of the unipolar subtype, right handedness, and age within the range of 13–18 years old. Exclusion criteria included positive history of head injury, systematic medical illnesses, psychotic disorders, mental retardation, autism, mania, alcohol and illicit substance abuse, eating disorders, and learning disability, as well as general exclusion criteria applicable for MRI scanning. The HC subjects had no personal or family history of psychiatric illness. All the adolescents had an intelligence quotient above 85 according to WISC-II [17]. Written informed consents were obtained from parents or guardians of all the subjects enrolled in this study. This research protocol was approved by the local Medical Ethics Committee in the Second Xiangya Hospital of Central South University, China.

### Psychiatric evaluations

The diagnosis of MDD was established by a structured interview which was conducted by two certified pediatric psychiatrists (JD, LS) according to the DSM-IV criteria [18]. The interview tool was the Development and Well-Being Assessment (DAWBA) [19], a package of questionnaires, interviews, and rating techniques designed to generate ICD-10 and DSM-IV psychiatric diagnoses for children ages 5 to 16 years old.

All the participating adolescents were rated by a short Mood and Feeling Questionnaire (SMFQ) [20] and a Screen for Child Anxiety Related Emotional Disorders (SCARED) [21] to assess the severity of their depression and anxiety symptoms. These two questionnaires have been translated into Chinese and tested for their reliability and validity [22], [23]. The SMFQ, which was designed to provide a rapid checklist of core symptoms for children aged 8–16 years old, is a 13-item self-descriptive scale. These 13 items included miserable or unhappy, didn't enjoy anything, tired, restless, no good, cried a lot, poor concentration, hated myself, bad person, lonely, unloved, never be as good, and did everything wrong [20]. A higher score indicates more serious degree of the depressive symptoms of the adolescent subject.

### MRI data acquisition and protocol

Imaging data were acquired using a 3 Tesla MRI system (Siemens, Germany) in the Department of Medical Imaging, Provincial People Hospital of Hunan. Subjects were instructed to relax with their eyes closed and keep their heads still during MRI scanning without falling asleep. They were asked after the MRI experiment if they had fallen asleep in the scanner. The data were excluded if the subject did not keep awake during the scan. Each participant was given earphones and a cushion for protection against scanner noise and minimization of any discomfort during MRI scanning. Anatomical images were first acquired using a T1-FL2D sequence (TR/TE = 350 ms/2.46 ms, matrix = 320*256, FOV = 24 cm*24 cm, slice thickness/gap = 4 mm/0.4 mm, 30 axial slices covered the whole brain) for image registration and functional localization. Resting-state fMRI images were then collected in the same slice orientation with a GRE-EPI sequence (TR/TE = 3000 ms/30 ms, FA = 90°, matrix = 64×64, FOV = 24 cm×24 cm, slice thickness/gap = 3.0 mm/0.3 mm) to yield 150 brain volumes lasting for 450 seconds (7.5 minutes).

### Image preprocessing

Imaging data were preprocessed using SPM2 (http://www.fil.ion.ucl.ac.uk/spm). The functional images underwent slice-timing correction and realignment for head motion correction. Data from the subjects whose head motion exceeded 1 mm or rotation exceeded 1° during scanning were excluded. The standard Montreal Neurological Institute (MNI) template provided by SPM was used for spatial normalization with a resampling voxel size of 2×2×2 mm^3^. The functional images were spatially smoothed with an FWHM of 4 mm. After linear trends were removed, the data were band-pass filtered between 0.01 and 0.08 Hz to remove the effects of very-low-frequency drift and high frequency noises by using REST software (V1.3, http://restfmri.net/).

### ALFF analysis

The ALFF analysis was carried out using the REST software. The calculation procedure was described in detail in elsewhere [24], [25], [26]. In brief, a filtered time series was transformed to the frequency domain with a fast Fourier transform, obtaining the power spectrum as a result. Because the power of a given frequency is proportional to the square of the amplitude of this frequency component, the square root was calculated at each frequency of the power spectrum and the averaged square root was obtained at each voxel. This averaged square root was taken as the ALFF measurement. For standardization, the ALFF of each voxel was further divided by the global mean of ALFF values. Then a standardized ALFF map of the whole brain was obtained.

To explore the ALFF differences between the MDD and HC groups, a two-sample t test was performed on the individually normalized ALFF map in a voxel-wise manner. Considering the sensitiveness of the resting state fMRI to the age changes, the age was regressed against the rapid structural and functional changes of developmental trajectory during adolescents [27], [28]. Monte Carlo simulation was employed to perform the correction for multiple comparisons using the REST AlphaSim program [29]. In this study, a corrected significance level of *p*<0.05 was obtained by combining individual voxel probability threshold *p*<0.03 and a minimum cluster size of 179 voxels.

The regions showed significant alteration of ALFF values between the HC and MDD groups were treated as a mask for defining the regions of interest (ROIs). In consideration of the small sample size in the present study, ‘leave-one-out’ (LOO) comparison was used to assess the reproducibility and robustness of the regions showing group differences in ALFF [30]. Specifically, group analysis in ALFF was accomplished with one MDD participant left out (17 MDD and 18 HC subjects) every time. This procedure was repeated 18 times. Then 18 separate LOO maps were obtained. The threshold used in each of the separate LOO maps was same as that of the group analysis map with all MDD subjects included (group ALFF map). The reproducibility of each ROI in the group ALFF map was examined by sign test. A ROI was considered to be replicable well if it was inflated relative to the separated LOO-defined ROI in 13 of 18 cases (*p*<0.05).

The averaged resting BOLD signal over the voxels in each ROI was considered as the time course of the ROI. The ALFF values of each ROI were averaged across subjects in both the HC and MDD groups. In order to represent the resting brain activity level for a neural (sub) system, the ALFF values of the ROIs in the frontal regions and subcortical structures were averaged respectively in each group, which was designated as the Frontal ALFF and the Subcortical ALFF. In each group, the difference between the Frontal ALFF and the Subcortical ALFF was assessed paired t test. The difference of the Frontal (Subcortical) ALFF between the HC and MDD groups was detected by two-sample t test. The regional ALFF difference between frontal and subcortical system was expressed by the subtraction of Frontal ALFF and Subcortical ALFF. Such a difference between the HC and MDD groups was also evaluated by two-sample t test.

It was found that some brain areas with high physiological noise, such as cistern areas, may show significant higher ALFF [24]. As an improved approach to detect the amplitude of low-frequency fluctuation for resting-state fMRI, fractional ALFF (fALFF) may suppress the signals of the cistern areas and is more sensitive to detect the neuronal activity. However, the measure of ALFF has higher reliability than fALFF [26]. For the purpose of the complementarily and verification of the result of ALFF, the difference of fALFF between the MDD and HC groups was also evaluated in the present study. The measure of fALFF is defined as the ratio of power spectrum of low-frequency (0.01–0.08 Hz) range to that of the entire frequency range was computed [31]. It may be regarded as the normalization of the ALFF. The fALFF analysis was carried out using the REST software. The difference of fALFF between the MDD and HC groups were evaluated by two-sample t test on the individually fALFF map in a voxel-wise manner with age regressed out.

### Correlation analysis

For each ROI, a correlation coefficient was calculated to assess the association between the ALFF values and the SMFQ and SCARED scores in MDD adolescents with age regressed out.

### Statistics analysis

The independent-sample t test and the chi-square test were used to compare the demographic data and the SMFQ and SCARED scores between two groups using SPSS 11.5 software (SPSS Inc., Chicago, IL, USA). The results were shown in the mean ± standard deviation format.

## Results

### Demographic and Clinical Comparisons

There were no significant differences between HC and the MDD groups in the ages and years of education of the subjects. The two groups differed significantly in the SMFQ and SCARED scores (Table 1). No subject had fallen asleep and was excluded due to head motion larger than 1 mm or rotation more than 1° during scanning.

**Table 1:** Demographic characteristics and clinical variables.

Characteristics	MDD (n = 18)	HC (n = 18)	*p* value
Gender (male/female)	8/10	8/10	0.99^a^
Age (years)	15.78 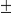 1.20	16.2 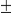 0.9	0.413^a^
Education years	9.2/1.1	9.5/0.8	0.311^b^
Illness Duration	<6 months	n/a	
SMFQ Score	16.67 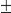 5.44	3.56 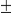 3.55	<0.001^b^
SCARED Score	44.39±19.10	14.89±10.61	<0.001^b^

Abbreviations:MDD, Major Depressive Disorder. HC, Healthy Control group. SMFQ, Short Mood and Feeling Questionnaire. SCARED: Screen for Child Anxiety Related Emotional Disorders. n/a: not applicable.

a The *p* value was obtained by Pearson χ2 two-tailed test;

b The *p* value was obtained by a two-sample two-tailed t test.

### ALFF differences

**Figure 1:**
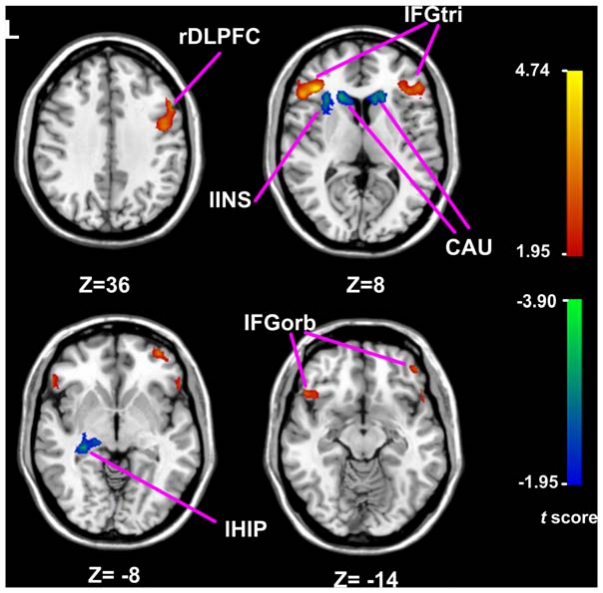
T-statistical map of the resting-state brain activity levels between the adolescents with MDD and HCs. The color-coded *t*-score bars indicated increased (warm color) ALFF and decreased (cold color) ALFF in the MDD patients relative to HCs. The voxels with *p*<0.03 and a cluster size of >179 were used to identify the clusters with significant differences; these criteria met a threshold of *p*<0.05 (corrected for multiple comparisons). The details of these regions were presented in Table S1.

After LOO comparison, 9 brain regions in the group ALFF map were inflated relative to the separate LOO maps more than 13 times (sign test, *p*<0.05). Compared to the HC group, the ALFF increased in the MDD group in five regions including the right dorsolateral frontal cortex (rDLPFC), bilateral inferior frontal gyrus (IFG) at the triangular region (IFGtri) and the orbital region (IFGorb). These regions are collectively referred to as the frontal regions and constitute the frontal system (Figure 1). In contrast, decreased ALFF was found in MDD adolescents in some subcortical regions including the left insular (lINS), bilateral caudate (CAU) and left hippocampus (lHIP). As the 4 ROIs mainly involve the para-limbic region (INS), limbic region (HIP) and striatum (CAU), they were also referred to as the (para) limbic-striatal system in this study. The details about the regions were shown in Table S1. The fMRI signals of these twelve regions of interest were extracted for further ROI-based ALFF analysis.

**Figure 2:**
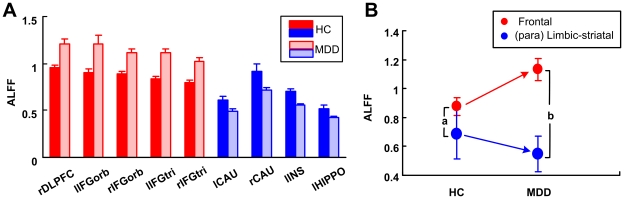
Brain ctivity imbalance in the fronto-subcortical activities shown by ALFF during resting-state. **A:** The mean ALFF of each ROI defined on Figure 1 for the HC and MDD groups was obtained by averaging across the HC subjects (n = 18; solid bar) and MDD patients (n = 18; dashed bar) in the frontal regions (red) and (para) limbic-striatal regions (blue). Error bars denoted the standard deviation of the mean ALFF across the subjects. **B:** The imbalance of fronto-(para) limbic striatal activities at a system level. The mean ALFF values of the ROIs in the frontal and the (para) limbic-striatal systems were further averaged to obtain the ALFF of the two systems (red: frontal, blue: (para) limbic-striatal). Error bars denote standard deviation of the mean ALFF across ROIs. The difference between the ALFF of the two systems in the HC and MDD group were shown by the letter of ‘a’ and ‘b’ respectively, whose numerical value are 0.189 and 0.585. This figure also demonstrated inter-group comparisons. From the HC to MDD, the frontal ALFF exhibits an increased trend (red arrow), whereas the (para) limbic-striatal ALFF exhibited a decreased trend (blue arrow).

The mean ALFF values of the ROIs were presented respectively using color bars (Figure 2A). Compared with the HC group, the MDD group showed higher ALFF in the frontal regions (red bars) and lower ALFF in (para) limbic-striatal regions (blue bars). The difference between frontal ALFF and (para) limbic-striatal ALFF was used to express the imbalance of the brain activity between the frontal and (para) limbic-striatal systems in resting state (Figure 2B). These mean values of the frontal ALFF demonstrated significantly greater values than the (para) limbic-striatal ALFF (*p*<0.0001 for both comparisons) in both the MDD and HC groups. The difference between the frontal ALFF and (para) limbic-striatal ALFF in the HC and MDD groups was 0.189 and 0.585 respectively (‘a’ and ‘b’ in Figure 2B). This ALFF difference between the two systems was significantly different between the HC and MDD groups (two sample t test, *p*<0.0001). The inter-group comparisons revealed an increased trend of the frontal ALFF (red arrow), and a decreased trend (blue arrow) of the (para) limbic-striatal ALFF in MDD adolescents (Figure 2B). Furthermore, the frontal ALFF and (para) limbic-striatal ALFF were both significantly different between the MDD and the HC groups (two sample t test, *p*<0.0001 for both comparisons). Brain regions showed difference of ALFF coincidently had similar fALFF changes between the MDD and HC groups. The t-statistical map of fALFF was shown in Figure S1.

No significant correlation was found between the ALFF values and SMFQ as well as SCARED scores in MDD adolescents. The results of correlation analysis with and without age being regressed out were shown in Table S2 and Table S3.

## Discussion

The present study showed that resting brain activity levels are higher in the frontal cortex than in the subcortical system comprised mainly of (para) limbic-striatal regions in both HC and MDD adolescents. The imbalance of brain activity between frontal and subcortical systems was increased in MDD adolescents as compared to the controls.

Psychological and psychiatric disorders, such as MDD, are traditionally diagnosed and studied mainly according to their clinical features and behavior characterizations. The application of MRI renders the opportunities for functional brain activity analysis. A number of relevant fMRI methodologies have been established. These new methods may all find applications in functional brain research, which built the base for modern functional imaging studies. However, each of these methodologies may have its own advantages and disadvantages. In recent years, the analysis of spontaneous brain activities during resting state has become an important tool for investigating neural mechanisms underpinning neuropsychiatric disorders. Practically, the resting brain activity can be quantified by the low-frequency (0.01–0.08 Hz) fluctuation of BOLD signals that has been used as a neurophysiological index [32] and reflect spontaneous neural activity of the brain [33]. The ALFF, measured by the total power within the frequency band of 0.01 to 0.08 Hz [24], provides information about synchronous cerebral activity and has been widely used in the study of both normal and pathological brain functions [26], [33], [34]. The present study is an effort in employing this method in adolescent MDD, and found some results that may have implications in its diagnosis and treatment.

Estimated by voxel-wised ALFF, different resting-state brain activity levels were identified between brain regions and between MDD and healthy adolescents (Figure 1). Both increased and decreased resting-state brain activity levels were found in MDD adolescents. Similar findings were obtained when these brain activities were analyzed using ROI-based ALFF: the frontal cortex manifests a higher resting magnitude of neural activity under baseline condition while the (para) limbic-striatal regions show lower resting magnitudes (Figure 2A). These altered resting-state brain activities were confirmed by at the systematic level (Figure 2B). The higher neural activity of frontal cortex may reflect a larger cognitive effort required to exert effective inhibitory control over subcortical regions in adolescents. Factors necessitating this increased cognitive effort include immature synaptic pruning [35], damage fiber tracts connecting the two systems, and other anatomical considerations. In MDD patients the difference of brain activity between the frontal cortex and (para) limbic-striatal regions is significantly increased as compared with the HCs (Figure 2). Thus, these data suggest that in the resting state the MDD adolescents may have further reduced capability or need more effort to regulate emotional processes.

Though the neurobiological processes of the same psychiatric illness is different between adolescents and adults because of the immaturity of the neural networks that mediate emotion processing during adolescence [36], adolescent MDD may be regarded as a strong predictor of MDD in adulthood [1]. Comparative studies between the adolescents and adults may provide more information useful for the development and prevent of the MDD. Functional imaging analyses of the adult MDD have shown alterations of these frontal and subcortical regions. For example, the orbital frontal cortex (OFC) has increased metabolism or blood flow in resting state in young relative to middle-aged samples [37], [38], [39]. Treatment with deep brain stimulation produced decreased metabolism of the OFC [40]. The decreased metabolism of right caudate and left putamen has been reported in adult MDD patients [40], [41]. Our results were consistent with these findings. Nevertheless, there is conflicting evidence regarding the resting-state alterations of these frontal and subcortical regions. Some studies reported the hypometabolism of dorsal prefrontal cotex [42], a greater baseline metabolism in caudate [43] or no significant differences in the subcortical regions [44]. Resting state fMRI reported an increased Regional homogeneity (ReHo) in putamen and frontal cortex [45], and a decreased ReHo in right orbitofrontal cortex and right insula [46]. These discrepant findings between the adolescent and adult MDD patients may be attributed to the methodologies used, participant selected, such as age range, depression severity, medication status, and family history in different studies.

The DLPFC plays an essential role in mood regulation, decision making, and working memory [47]. The DLPFC is the last brain area to begin myelination and may be the only area that continues myelination throughout human life [48]. DLPFC abnormalities have been observed in depressed adolescents [49]. The DLPFC has often been used as the target site of repetitive transcranial magnetic stimulation (TMS) for treatment of medication-resistant depression [50]. In the present study, the brain activity of rDLPFC was found to be the highest among the 9 brain regions in both the MDD and HC groups (Figure 2A). The result strengthened the key role of DLPFC in the neuropathology of adolescent MDD in the views of the local brain activity level.

In summary, our results demonstrated an increased imbalance of resting-state brain activity between the frontal system and (para) limbic-striatal system MDD adolescents. These data may suggest an inability to regulate subcortical emotional processes by the frontal executive system despite increased efforts. These findings provide insights into the neural correlates of MDD and may lead to strategies for its prevention and treatment, such as the rTMS treatment for depression.

## Supporting Information

Figure S1 **T-statistical map of fALFF between the adolescents with MDD and HC groups.** The color-coded *t*-score bars indicated increased (warm color) fALFF and decreased (cold color) fALFF in the MDD patients relative to HCs. The voxels with *p*<0.01 and a cluster size of >10 were used to identify the clusters with significant differences (uncorrected).
(TIF)

Table S1 **Regions showing ALFF differences between MDD and HC groups.** Abbreviations: l: left. r: right. DLPFC: dorsolateral prefrontal cortex. IFGtri: triangular inferior frontal gyrus. IFGorb: orbital inferior frontal gyrus. CAU: caudate. INS: insular. HIP: hippocampus. BA: Brodmann's area; Volume = number of clusters. MNI: Montreal Neurological Institute Coordinate System or Template; *t*: statistical value of peak voxel showing ALFF differences between the two groups.
(DOC)

Table S2 **Correlation between ROI ALFF values and behavioral scores without age regressed out.** Correlation coefficient (up) and p values (low) were shown.
(DOC)

Table S3 **Correlation between ROI ALFF values and behavioral scores with age regressed out.** Correlation coefficient (up) and p values (low) were shown.
(DOC)
