# Supplementary figures and images for: Increased Activity Imbalance in Fronto-Subcortical Circuits in Adolescents with Major Depression
Source: PLoS One. 2011 Sep 16;6(9):e25159. doi: 10.1371/journal.pone.0025159 (PMC3175001; doi:10.1371/journal.pone.0025159)

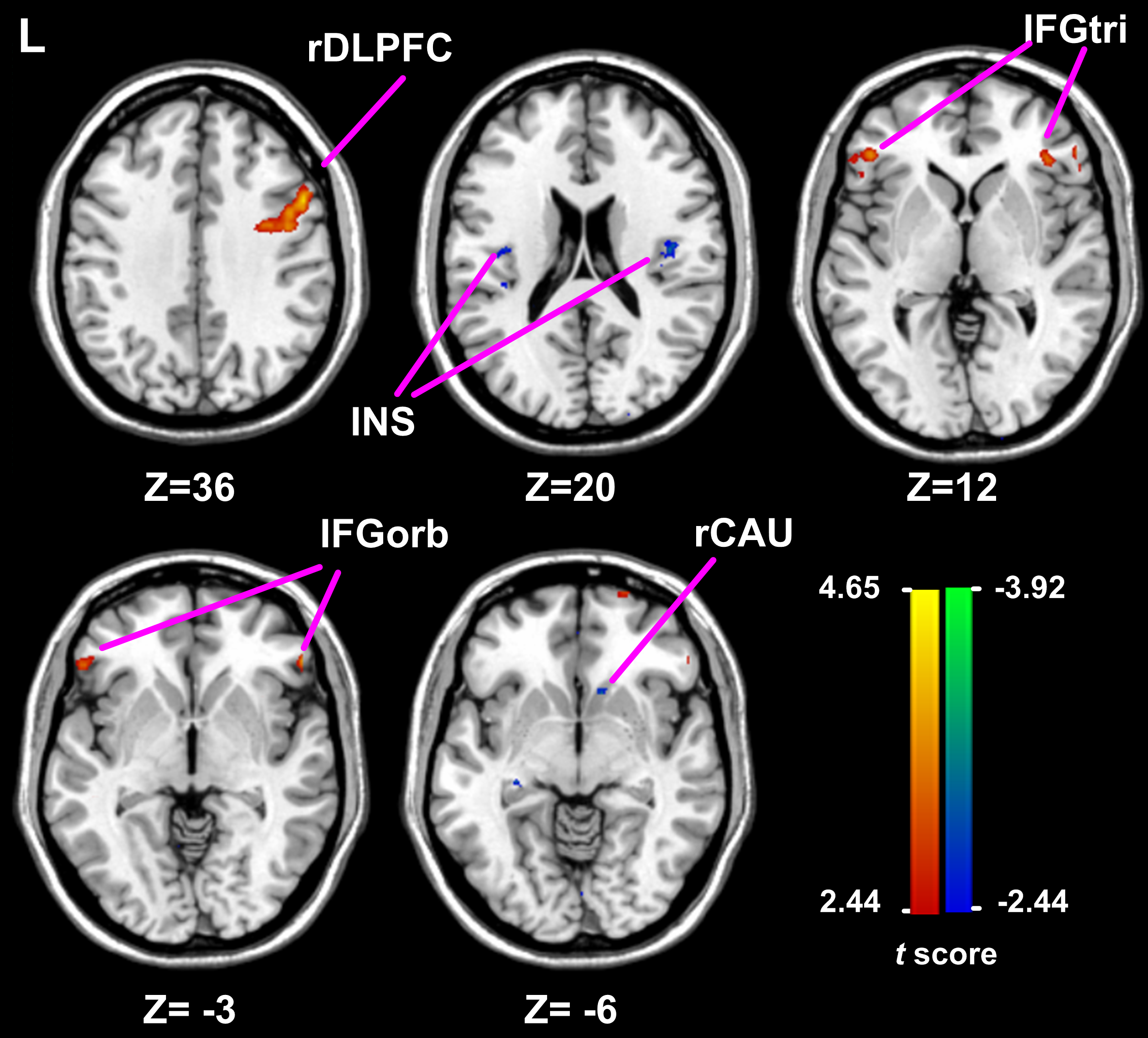

Supplement: Figure S1 — T-statistical map of fALFF between the adolescents with MDD and HC groups. The color-coded t-score bars indicated increased (warm color) fALFF and decreased (cold color) fALFF in the MDD patients relative to HCs. The voxels with p<0.01 and a cluster size of >10 were used to identify the clusters with significant differences (uncorrected). (TIF) [file pone.0025159.s001.tif]
