# Supplementary material for: Increased Activity Imbalance in Fronto-Subcortical Circuits in Adolescents with Major Depression
Source: PLoS One. 2011 Sep 16;6(9):e25159. doi: 10.1371/journal.pone.0025159 (PMC3175001; doi:10.1371/journal.pone.0025159)
Supplement: Table S1 — Regions showing ALFF differences between MDD and HC groups. Abbreviations: l: left. r: right. DLPFC: dorsolateral prefrontal cortex. IFGtri: triangular inferior frontal gyrus. IFGorb: orbital inferior frontal gyrus. CAU: caudate. INS: insular. HIP: hippocampus. BA: Brodmann's area; Volume = number of clusters. MNI: Montreal Neurological Institute Coordinate System or Template; t: statistical value of peak voxel showing ALFF differences between the two groups. (DOC) [file pone.0025159.s002.doc]

**Table S1. Regions showing ALFF differences between MDD and HC groups**.

| Brain regions | BA | MNI Coordinates | | | Maximum | Volume |
| --- | --- | --- | --- | --- | --- | --- |
|  |  | *x* | *y* | *z* | *t* value |  |
| Frontal |  |  |  |  |  |  |
| lIFGtri | 47 | 40 | 32 | 8 | 4.73 | 659 |
| rDLPFC | 9 | -48 | 4 | 36 | 3.48 | 405 |
| rIFGtri | 47 | -42 | 30 | 8 | 2.90 | 588 |
| rIFGorb | 47 | -52 | 28 | -4 | 2.26 | 144 |
| lIFGorb | 47 | 54 | 28 | -4 | 2.11 | 68 |
| (para) Limbic-striatal |  |  |  |  |  |  |
| rCAU |  | -12 | 22 | 10 | -4.07 | 74 |
| lINS | 13 | 32 | 20 | 8 | -2.99 | 125 |
| lCAU |  | 14 | 22 | 6 | -2.86 | 162 |
| lHIP |  | 36 | 24 | -12 | -2.02 | 63 |

Abbreviations: l: left. r: right. DLPFC: dorsolateral prefrontal cortex. IFGtri: triangular inferior frontal gyrus. IFGorb: orbital inferior frontal gyrus. CAU: caudate. INS: insular. HIP: hippocampus. BA: Brodmann's area; Volume = number of clusters. MNI: Montreal Neurological Institute Coordinate System or Template; *t*: statistical value of peak voxel showing ALFF differences between the two groups.
