# Supplementary material for: Increased Activity Imbalance in Fronto-Subcortical Circuits in Adolescents with Major Depression
Source: PLoS One. 2011 Sep 16;6(9):e25159. doi: 10.1371/journal.pone.0025159 (PMC3175001; doi:10.1371/journal.pone.0025159)
Supplement: Table S2 — Correlation between ROI ALFF values and behavioral scores without age regressed out. Correlation coefficient (up) and p values (low) were shown. (DOC) [file pone.0025159.s003.doc]

**Table S2. Correlation between ROI ALFF values and behavioral scores without age regressed out.**

**Correlation coefficient (up) and p values (low) were shown.**

|  | rDLPFC | lIFGorb | rIFGorb | lIFGtri | rIFGtri | lCAU | rCAU | lINS | lHIP |
| --- | --- | --- | --- | --- | --- | --- | --- | --- | --- |
| SMFQ | 0.279  (0.26) | -0.168  (0.504) | 0.001  (0.996) | -0.336  (0.172) | 0.107  (0.671) | -0.131  (0.603) | -0.122  (0.628) | 0.159  (0.527) | 0.162  (0.519) |
| SCARED | 0.319  (0.196) | -0.125  (0.620) | 0.111  (0.660) | -0.095  (0.704) | 0.392  (0.107) | -0.015  (0.951) | -0.062  (0.806) | 0.205  (0.412) | 0.445  (0.063) |
