# Supplementary material for: Increased Activity Imbalance in Fronto-Subcortical Circuits in Adolescents with Major Depression
Source: PLoS One. 2011 Sep 16;6(9):e25159. doi: 10.1371/journal.pone.0025159 (PMC3175001; doi:10.1371/journal.pone.0025159)
Supplement: Table S3 — Correlation between ROI ALFF values and behavioral scores with age regressed out. Correlation coefficient (up) and p values (low) were shown. (DOC) [file pone.0025159.s004.doc]

**Table S3. Correlation between ROI ALFF values and behavioral scores with age regressed out.**

**Correlation coefficient (up) and p values (low) were shown.**

|  | rDLPFC | lIFGorb | rIFGorb | lIFGtri | rIFGtri | lCAU | rCAU | lINS | lHIP |
| --- | --- | --- | --- | --- | --- | --- | --- | --- | --- |
| SMFQ | 0.319  (0.195) | -0.192  (0.443) | -0.007  (0.976) | -0.372  (0.127) | 0.113  (0.652) | -0.161  (0.521) | -0.120  (0.633) | 0.162  (0.520) | 0.157  (0.531) |
| SCARED | 0.311  (0.207) | -0.106  (0.672) | 0.125  (0.618) | -0.074  (0.767) | 0.385  (0.113) | 0.014  (0.953) | -0.065  (0.797) | 0.202  (0.421) | 0.456  (0.056) |
